# Correction: Miu et al. MRC-5 Human Lung Fibroblasts Alleviate the Genotoxic Effect of Fe-N Co-Doped Titanium Dioxide Nanoparticles Through an OGG1/2-Dependent Reparatory Mechanism. *Int. J. Mol. Sci.* 2023, *24*, 6401

**DOI:** 10.3390/ijms27052347

**Published:** 2026-03-03

**Authors:** Bogdan Andrei Miu, Ionela Cristina Voinea, Lucian Diamandescu, Anca Dinischiotu

**Affiliations:** 1 Department of Biochemistry and Molecular Biology, Faculty of Biology, University of Bucharest, 91-95 Splaiul Independentei, 050095 Bucharest, Romania; bogdan-andrei.miu@bio.unibuc.ro (B.A.M.); anca.dinischiotu@bio.unibuc.ro (A.D.); 2 Research Institute of the University of Bucharest—ICUB, University of Bucharest, 050657 Bucharest, Romania; 3 National Institute of Materials Physics (NIMP), Atomistilor 405A, 077125 Magurele, Romania; diamand@infim.ro

There was an error in the original publication. In the original publication [1], Figure 2, “Observation of TiO_2_ NPs size and morphology”, was based on previously published nanoparticles’ characterization data of the authors’ research group [75] and was included in the manuscript during the peer-review process following the necessity of illustrative physicochemical data. Figure 2 and its legend have been removed from the publication.

Accordingly, a correction has been made to Section 2.1, “Physicochemical Characteristics of TiO_2_ NPs”, where the following paragraph (4) was removed:

As revealed in the images obtained by transmission electron microscopy (TEM), both types of NPs generally had near polyhedral shapes with round corners (Figure 2a,b); some spheres could also be observed. The dimension of most P25 NPs was between ∼10 and 50 nm and had a mean particle size of 29 nm (Figure 2a). The size range of P25-Fe(1%)-N NPs was larger, with most being ∼15–60 nm. However, the mean particle size of P25-Fe(1%)-N NPs was similar to that of P25 NPs, i.e., ∼28 nm (Figure 2b). More analyses regarding the characterization of TiO_2_ NPs were provided in our previously published papers [70,75].

In addition, a correction has been made to Section 4.1, “Physicochemical Characterization of TiO_2_ NPs”, where the following sentence from paragraph 2 was removed:

TEM images and measurements were performed on a JEOL 200 CX transmission electron microscope (accelerating voltage: 200 kV).

With this correction, the order of some references has been adjusted accordingly. The authors state that the scientific conclusions are unaffected. This correction was approved by the Academic Editor. The original publication has also been updated.
